# Safety and Efficacy of Iron Chelators in Patients With Chronic Kidney Disease: A Systematic Review and Meta‐Analysis

**DOI:** 10.1111/hdi.70103

**Published:** 2026-06-18

**Authors:** Humam Emad Rajha, Richard Shamoon, Ameer Almeshagbeh, Samah Masry, Fadel Alqatati, Baha H. Abuajameia, Abdulqadir J. Nashwan

**Affiliations:** ^1^ College of Medicine, QU Health, Qatar University Doha Qatar; ^2^ Nursing and Midwifery Research Department Hamad Medical Corporation Doha Qatar; ^3^ Department of Molecular, Cellular, and Developmental Biology University of Michigan Ann Arbor Michigan USA; ^4^ Department of Internal Medicine Hamad Medical Corporation Doha Qatar

**Keywords:** chronic kidney disease, deferasirox, deferoxamine, iron chelation, iron overload, meta‐analysis, systematic review

## Abstract

**Background:**

Iron overload in chronic kidney disease contributes to oxidative injury. The role of iron chelators, standard in transfusion‐related overload, remains unclear in chronic kidney disease.

**Aim:**

To evaluate the efficacy and safety of iron chelators in adults with chronic kidney disease.

**Methods:**

A systematic review and meta‐analysis followed PRISMA 2020 guidelines. Databases were searched for randomized controlled trials and observational studies reporting on iron indices, hematological parameters, and adverse events.

**Results:**

Eleven studies (206 patients) were included. Iron chelation significantly reduced serum ferritin (weighted mean difference [WMD]: −1.48 μg/L; 95% CI: −2.09, −0.87), transferrin (WMD: −10.6 mg/dL; 95% CI: −15.6, −5.61), and serum iron (WMD: −24.2 μg/dL; 95% CI: −42.5, −5.9). Hemoglobin increased (WMD: 1.07 g/dL; 95% CI: 0.20, 1.94). Glucose levels decreased, but lipid parameters were unaffected. Evidence was limited by high heterogeneity and potential bias. Gastrointestinal adverse events were common; serious events were rare.

**Conclusion:**

Iron chelation effectively reduces iron stores and may improve anemia in chronic kidney disease patients. However, the evidence is limited by small sample sizes and heterogeneity. High‐quality randomized controlled trials are needed to establish clinical utility and safety before routine use.

## 1 Introduction

Chronic kidney disease (CKD) is a substantial global health burden, affecting more than 10% of the world's population and contributing to progressive complications along with a heightened risk of premature death [1, 2]. Among its numerous complications, anemia is one of the most prevalent and clinically consequential. Accordingly, management typically includes erythropoiesis‐stimulating agents (ESAs) and iron supplementation aimed at correcting the anemia [3]. Nevertheless, frequent intravenous iron administration and transfusion therapy, although often necessary, predispose patients to iron overload. This condition is marked by the pathological accumulation of iron in vital organs, including the liver, heart, and endocrine glands [4].

Iron overload is a detrimental condition with significant clinical implications. It has been reported in up to 28.6% of patients with advanced CKD on peritoneal dialysis, compared with as high as 75% in those receiving hemodialysis, underscoring its clinical relevance in this population [5, 6]. According to US claims data, iron overload in CKD patients was associated with an incremental economic burden of nearly $44,000 per patient annually, far exceeding the additional costs observed in other chronic comorbidities [7]. Excessive deposition of iron drives oxidative stress, triggering tissue injury that may manifest in complications such as hepatic fibrosis, cardiomyopathy, arrhythmias, heart failure, and increased susceptibility to infection [4, 8]. These sequelae exacerbate the already high morbidity and mortality burden in CKD, underscoring the need for effective strategies to mitigate iron toxicity.

Iron chelation therapy is the cornerstone of systemic iron overload management. Chelating agents work by binding circulating and tissue‐deposited iron, forming complexes that are subsequently excreted, thereby reducing oxidative injury [9, 10]. Currently, three chelators are in clinical use, namely deferoxamine, a parenterally administered chelator, and the oral formulations deferasirox and deferiprone. Through sequestration of labile and stored iron, chelators lower the availability of redox‐active iron and mitigate the toxic consequences of oxidative stress [11]. This therapeutic benefit is well established in hereditary and transfusion‐dependent disorders such as thalassemia, where chelators have demonstrated effectiveness in lowering ferritin and enhancing long‐term outcomes [12].

In CKD, however, the role of iron chelation remains uncertain. Patients with CKD differ from those with transfusion‐dependent disorders. Chronic inflammation, dysregulated hepcidin, and impaired erythropoiesis alter iron metabolism. Reduced renal clearance may also limit drug elimination. As a result, the normal balance between iron storage, release, and utilization is disrupted, complicating the management of anemia and iron overload [13]. These factors raise concerns about whether chelators are effective and whether they may cause harm. Additional risks include nephrotoxicity, gastrointestinal intolerance, and rare but severe adverse events such as hepatic or pancreatic injury. Because of these uncertainties, clinicians often extrapolate from evidence in hematological populations such as patients with thalassemia [14, 15, 16, 17]. This leaves nephrologists without clear clinical guidelines that provide recommendations for the use of iron chelation in CKD, contributing to wide variability in practice. Therefore, a systematic synthesis of the available evidence is therefore needed to clarify the risks and benefits of iron chelation in this high‐risk group.

To address this knowledge gap, we conducted a systematic review and meta‐analysis to evaluate the efficacy and safety of iron chelation therapy in CKD. For this purpose, we drew on evidence from randomized trials and observational studies to quantify the effect of iron chelators on iron indices (serum ferritin, transferrin saturation, and serum iron), hematological parameters (hemoglobin and hematocrit), and metabolic outcomes (glucose and lipid profile), while documenting the range of reported adverse events. By consolidating available evidence, this study seeks to provide an evidence‐based framework to inform clinical decision‐making and guide future research into the role of iron chelation therapy in CKD.

## 2 Methods

### 2.1 Protocol and Registration

This systematic review and meta‐analysis was conducted and reported in accordance with the Preferred Reporting Items for Systematic Reviews and Meta‐Analyses (PRISMA) 2020 guidelines [18]. The review protocol was prospectively registered in the International Prospective Register of Systematic Reviews (PROSPERO) (Registration ID: CRD420251103586).

### 2.2 Search Strategy and Data Sources

A comprehensive search was developed and conducted on June 23, 2025, across six electronic databases: PubMed, Cochrane Central Register of Controlled Trials (CENTRAL), Cumulative Index to Nursing and Allied Health Literature (CINAHL), Scopus, EMBASE, and Web of Science. The search strategy combined controlled vocabulary (e.g., MeSH, Emtree) and free‐text keywords related to the concepts of “chronic kidney disease,” “renal failure,” “dialysis,” “renal replacement therapy,” and specific “iron chelators” (including deferoxamine, deferasirox, and deferiprone). No restrictions were applied on language or publication date. The full search strategy for all databases is available in Table S1. Furthermore, reference lists of included studies were manually screened (citation tracking) to identify additional eligible reports. Searches of trial registries and gray literature were also planned.

### 2.3 Eligibility Criteria

Studies were considered eligible if they enrolled adult patients (≥ 18 years) with a diagnosis of CKD, irrespective of dialysis status. Eligible interventions included the administration of a specific iron chelator (deferoxamine, deferasirox, or deferiprone), either as monotherapy or compared against a control group (placebo, no intervention, or standard therapy without chelation). Studies were required to report on at least one predefined efficacy or safety outcome, including: iron indices (serum ferritin, transferrin saturation, and serum iron), hematological parameters (hemoglobin and hematocrit), metabolic outcomes (glucose and lipid profile), and safety outcomes (any side effects). Only original research articles with an analytical study design, including randomized controlled trials (RCTs) and observational studies (prospective, retrospective, or single‐arm pre–post designs), were included. Studies were limited to those published in English and available as a full‐text report. Exclusion criteria included non‐original publications (reviews, editorials, commentaries, letters), conference abstracts without accompanying full text, case reports or small case series (< 5 patients), preclinical or in vitro studies, studies conducted in non‐CKD populations, investigations that did not assess the specified iron chelators, and articles lacking extractable outcome data as defined in the inclusion criteria. Duplicate reports of the same study population were also excluded.

### 2.4 Study Selection and Screening

Search results from all databases were collated and uploaded to EndNote (Version 20, Clarivate Analytics) for deduplication. The deduplicated records were then imported into Rayyan systematic review software for screening (https://www.rayyan.ai/). Three independent reviewer pairs screened the titles and abstracts of all identified records against the eligibility criteria. Any discrepancies between reviewers were resolved through discussion or by a senior member of the research team. The full texts of all potentially relevant studies were retrieved and independently assessed for eligibility using pre‐defined inclusion and exclusion criteria. Primary reasons for exclusion during the full‐text screening phase were documented and recorded in a Microsoft Excel spreadsheet.

### 2.5 Outcomes

The primary outcomes were efficacy endpoints, including changes in iron indices (serum ferritin, transferrin saturation, and serum iron) and red blood cell measures (hemoglobin and hematocrit). Secondary outcomes included changes in metabolic parameters (lipid profile and glucose) and safety outcomes (drug‐related adverse events).

### 2.6 Data Extraction

Data were extracted independently by two pairs using a standardized Microsoft Excel template. Extracted variables included study details (author, year, country, design, and duration), participant characteristics (sample size, mean age, sex distribution, CKD stage, or dialysis status), intervention details (chelator type, dose, route, and duration), and reported outcomes. Discrepancies were resolved through discussion and by an expert opinion of a senior author. Additionally, Supporting Information and appendices of the published articles were thoroughly examined to identify any further relevant data.

### 2.7 Quality and Risk of Bias Assessment

The quality of the included studies was assessed using Risk of Bias 2.0 (RoB 2.0) [19] for RCTs and the Newcastle–Ottawa Scale (NOS) [20] for observational studies. RoB 2.0 was used to evaluate the risk of bias in RCTs, focusing on domains such as randomization, allocation concealment, blinding, and outcome reporting. For observational studies, the NOS was employed to assess quality by evaluating selection, comparability, and exposure or outcome assessment. Studies with NOS scores ≥ 7 were considered as having a low risk of bias, scores of 4–6 as having a moderate risk of bias, and scores < 4 as having a high risk of bias. Both tools were used to ensure a comprehensive evaluation of the internal validity of the included studies.

### 2.8 Data Synthesis and Statistical Analysis

A preliminary search confirmed a limited number of RCTs. Consequently, we made a post hoc decision to include prospective observational studies to provide a more comprehensive, albeit less definitive, summary of the current evidence. All included study designs are presented together to allow for a complete narrative and quantitative synthesis, with the understanding that this combines studies with different inherent risks of bias.

Characteristics of the included studies, as well as side effects of included studies, were presented in tables and narratively described. Continuous outcomes were pooled and expressed as weighted mean differences (WMDs) with 95% confidence intervals (CIs). WMD is a measure of the average difference between intervention and control groups weighted by study precision, and was used for continuous outcomes. A random‐effects model using DerSimonian–Laird (DL) estimation was applied for all meta‐analyses to account for anticipated clinical and methodological heterogeneity. Heterogeneity was assessed using the *I* ^2^ statistic and Cochran's *Q* test. *I* ^2^ values were interpreted using the following thresholds: 1%–24% low, 25%–49% moderate, 50%–74% high, and ≥ 75% substantial heterogeneity. The *τ* ^2^ statistic was used to estimate the between‐study variance. Pre‐specified subgroup analyses were planned to compare outcomes by chelator type, route of administration, and duration of iron chelator. While chelator‐type subgroups were done where possible, the other subgroup analyses were not possible due to the lack of sufficient data on these items. Sensitivity analyses were conducted sequentially, excluding studies deemed to be at high risk of bias, to test the robustness of the pooled results. Publication bias was assessed using Doi plots and the Luis Furuya‐Kanamori (LFK) index. Statistical analyses were conducted using Stata version 18 (StataCorp, College Station, TX, USA).

### 2.9 Ethics

This study utilized published data and therefore did not require ethical approval.

## 3 Results

### 3.1 Search Results (PRISMA)

Figure 1 illustrates the study selection process. The systematic search of six electronic databases (PubMed, Cochrane CENTRAL, CINAHL, Scopus, EMBASE, and Web of Science) identified a total of 1177 records. After removing duplicates, 621 unique records underwent title and abstract screening. At this stage, 554 studies were excluded as they did not meet the eligibility criteria. Full texts of 67 reports were sought for retrieval, of which 15 were not accessible. The 52 remaining reports assessed for eligibility were further evaluated, with 14 studies excluded due to incorrect study design, 11 due to an incorrect population, five due to the wrong intervention, two due to the wrong comparator, five due to the wrong outcome, and four due to language restrictions. Ultimately, 11 studies comprising 206 participants met the inclusion criteria and were included in the review.

**FIGURE 1:**
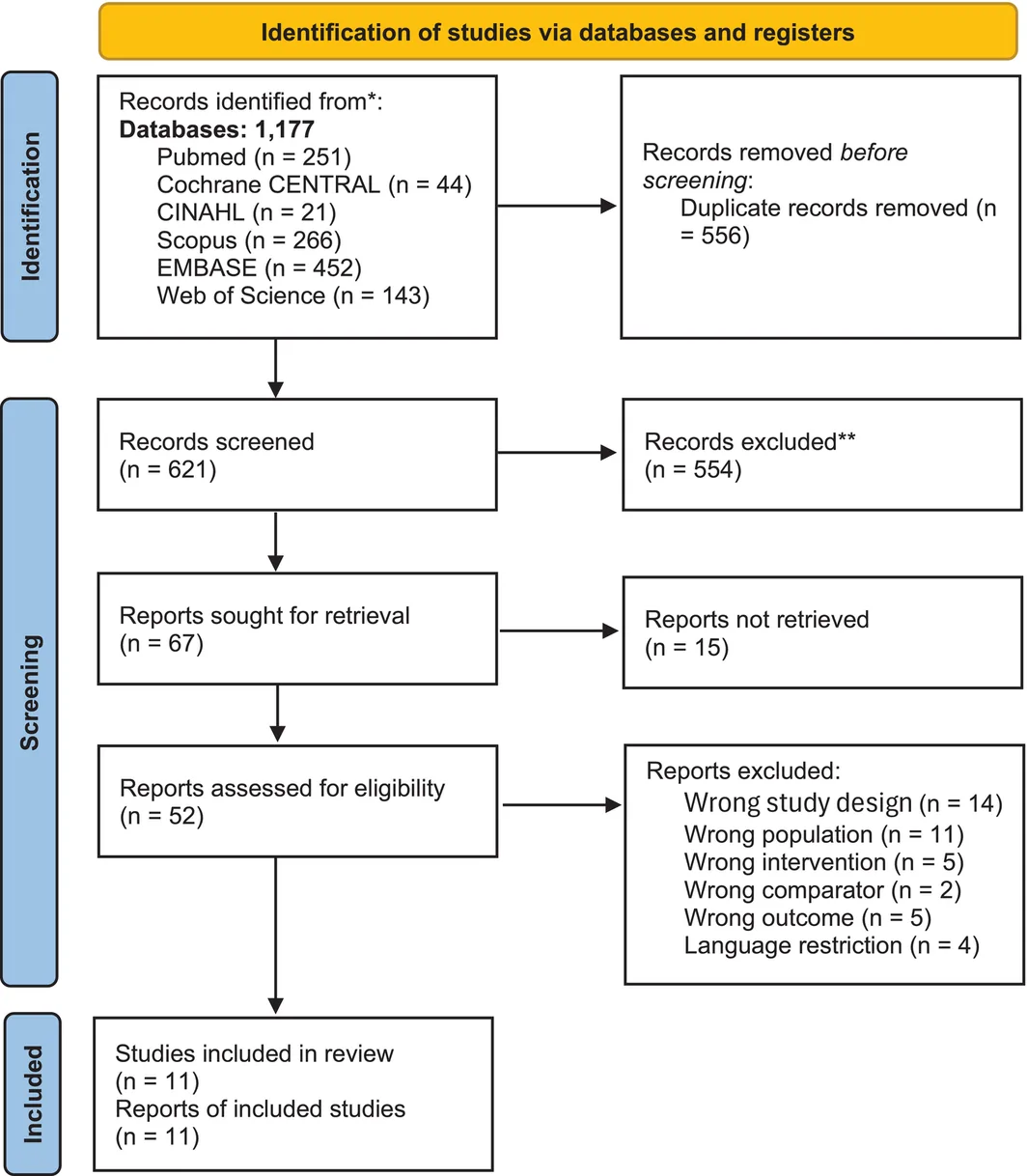
The PRISMA flowchart. * Identified and extracted and uploaded to rayyan systematic review software. ** No automation tool was used. All screening was done manually.

### 3.2 Characteristics of Included Studies

The characteristics of the 11 studies included in this review are summarized in Table 1. Of the included studies, three were RCTs, five were of prospective design, and three were retrospective in nature. These studies were published between 1988 and 2024 and conducted across diverse countries, including Mexico, Brazil, Algeria, Taiwan, Egypt, Italy, Spain, the US, and Belgium. The sample sizes ranged from seven to 54 participants, with the proportion of male participants varying from 25% to 72.2%. The mean study duration ranged from 6 to 84 months. The mean age of participants varied from 35.7 to 56 years, indicating predominantly adult populations. Among the included studies, three focused on oral Deferasirox, while the other eight investigated intravenous or intradialytic Deferoxamine.

**TABLE 1:** Characteristics of included studies.

References	Study design	Study duration	Country	Mean age (SD)	Male sex, *n* (%)	Sample size	Chelator type	Chelator route
Murillo Ortiz [21]	RCT	6 months	Mexico	44 (16.3)	39 (72.2%)	54	Deferasirox	Oral
Nunes et al. [22]	Prospective study	12 months	Brazil	56 (13)	17 (60.7%)	28	Deferoxamine	Intravenous
Bekhechi et al. [23]	Retrospective study	48 months	Algeria	48.2 (14.3)	12 (38.7%)	31	Deferasirox	Oral
Chen, Shu, and Yang [24]	Retrospective study	52 months	Taiwan	52.8 (15.4)	1 (12.5%)	8	Deferasirox	Oral
Alnahal et al. [25]	Prospective study	17 months	Egypt	48.4 (6.6)	6 (60%)	10	Deferoxamine	Intradialytic
Lee et al. [26]	Prospective study	12 months	Taiwan	50.8 (6.9)	4 (50%)	8	Deferoxamine	Intravenous
De Marchi and Cecchin [27]	RCT	12 months	Italy	51.6 (10.7)	9 (42.9%)	7	Deferoxamine	Intravenous
Praga et al. [28]	Prospective study	14 months	Spain	35.7 (14.4)	4 (57%)	7	Deferoxamine	Intravenous
Stivelman et al. [16]	Prospective study	12 months	US	37.4 (3.2)	2 (25%)	8	Deferoxamine	Intravenous
Tielemans et al. [29]	Retrospective study	84 months	Belgium	—	—	21	Deferoxamine	Intradialytic
De la Serna et al. [30]	RCT	74 months	Spain	37.6 (14.7)	12 (50%)	24	Deferoxamine	Intravenous

*Note:* Sample sizes were derived from cohorts of patients with eligibility criteria.

Abbreviation: RCT, randomized controlled trial.

### 3.3 Effect of Iron Chelators on Iron Measures in CKD

#### 3.3.1 Effect of Iron Chelators on Transferrin in CKD

Seven studies [21, 22, 24, 26, 27, 29] assessed the effect of iron chelators on transferrin in CKD patients (Figure 2). These studies were classified by chelator type to allow contrast of the effects of each chelator on transferrin. The analysis compared the transferrin levels before and after the administration of iron chelators. For the deferasirox group, there was an overall reduction in transferrin levels, with a significant WMD of −11.8 (95% CI: −17.3, −6.4). The overall weight contribution from this subgroup was 40.76% with no significant heterogeneity (*I* ^2^ = 0%), indicating a consistent effect across these studies. For the deferoxamine group, a lower but present overall reduction in transferrin levels was also observed with a nonsignificant WMD of −8.38 (95% CI: −17.5, 0.70). However, individual studies showed mixed effects, with De Marchi and Cecchin [27] and Lee et al. [26] showing no change and a slight increase in transferrin levels, respectively. The weight contribution from this subgroup was 59.2%, and a high heterogeneity was observed (*I* ^2^ = 69.9%). The overall meta‐analysis revealed a significant overall WMD of −10.6 (95% CI: −15.6, −5.61), with moderate heterogeneity (*I* ^2^ = 49.0%, *p* = 0.067), suggesting a moderate variation between studies. The Doi plot revealed no asymmetry (LFK index = −0.72), suggesting no risk of publication bias (Figure S1).

**FIGURE 2:**
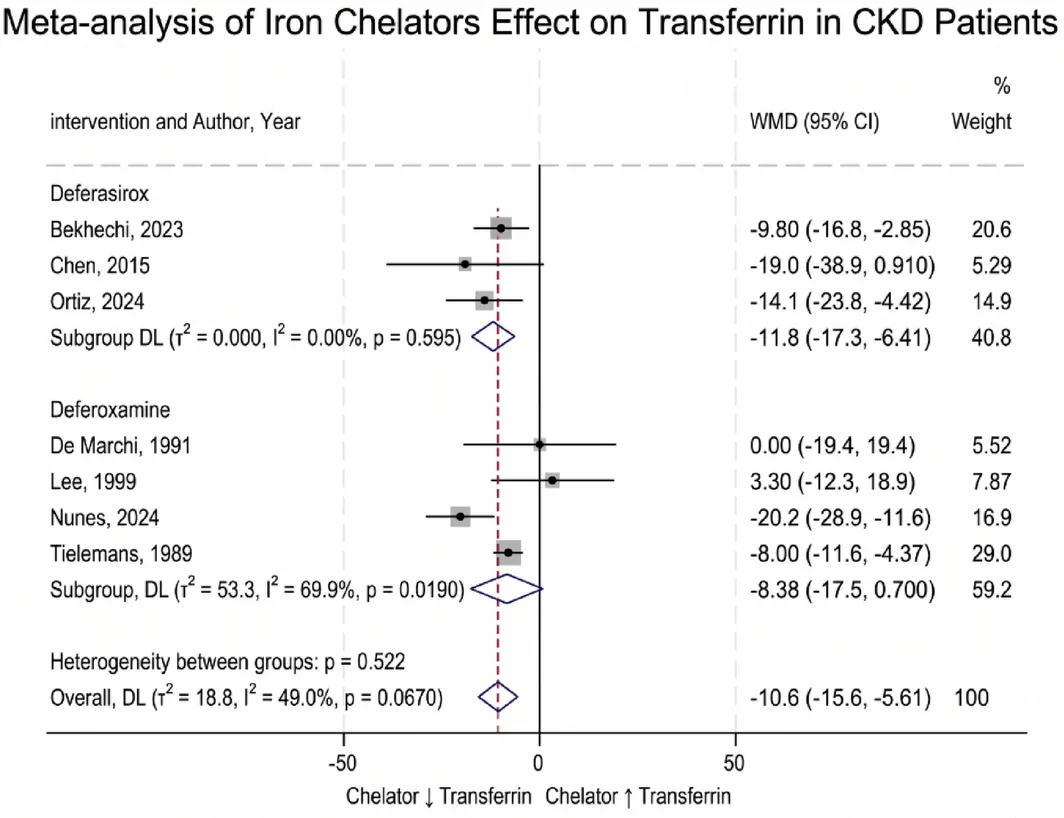
Forest plot of the effect of Iron chelators on transferrin in CKD patients. Weights and between‐subgroup heterogeneity test were derived from the random‐effects model.

#### 3.3.2 Effect of Iron Chelators on Ferritin in CKD

Eleven studies [16, 21, 22, 23, 24, 25, 26, 27, 28, 29, 30] assessed the effect of iron chelators on ferritin levels in CKD patients (Figure 3). These studies were categorized by chelator type to compare the effects of each chelator on serum ferritin. The analysis compared the serum ferritin levels before and after the administration of iron chelators. For the deferasirox group, there was an overall reduction in ferritin levels, with a significant WMD of −1.21 (95% CI: −1.76, −0.66). The overall weight contribution from this subgroup was 26.46% with moderate heterogeneity (*I* ^2^ = 38.5%). For the deferoxamine group, a more substantial reduction in ferritin levels was observed, with a WMD of −1.55 (95% CI: −2.28, −0.82). The weight contribution from this subgroup was 73.5% with substantial heterogeneity (*I* ^2^ = 97.6%, *p* < 0.001), due to individual studies such as Stivelman et al. [16] that showed a pronounced reduction in ferritin levels, while other studies, such as De Marchi and Cecchin [27] and Tielemans et al. [29] that presented a mixed effect. The overall meta‐analysis revealed a significant WMD of −1.48 (95% CI: −2.09, −0.87), with a substantial level of heterogeneity (*I* ^2^ = 96.8%). The Doi plot revealed major asymmetry (LFK index = −4.36), suggesting potential risk of major publication bias (Figure S2).

**FIGURE 3:**
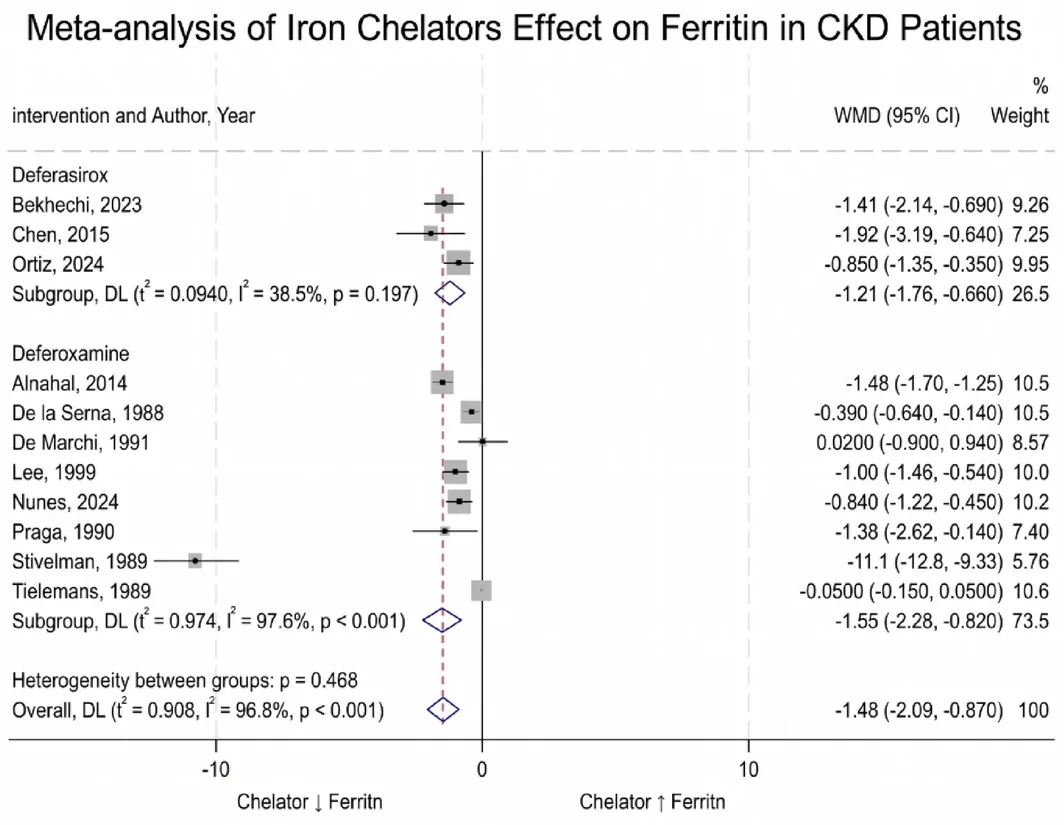
Forest plot of the effect of Iron chelators on ferritin in CKD patients. Weights and between‐subgroup heterogeneity test were derived from the random‐effects model.

#### 3.3.3 Effect of Iron Chelators on Serum Iron in CKD

The meta‐analysis of iron chelators' effect on serum iron levels in CKD patients included four studies [22, 23, 24, 29, 30] (Figure 4). The analysis compared the serum iron levels before and after the administration of iron chelators. The overall meta‐analysis revealed a significant WMD of −24.2 (95% CI: −42.5, −5.90) with high heterogeneity (*I* ^2^ = 63.5%, *p* = 0.042), indicating variability across studies. The analysis suggests that iron chelators generally reduce serum iron levels, although there is some heterogeneity in the findings, particularly due to the varying effects in individual studies such as Chen, Shu, and Yang [24], which showed an increase in serum iron after iron chelators. The Doi plot revealed major asymmetry (LFK index = +4.65), suggesting potential risk of major publication bias (Figure S3).

**FIGURE 4:**
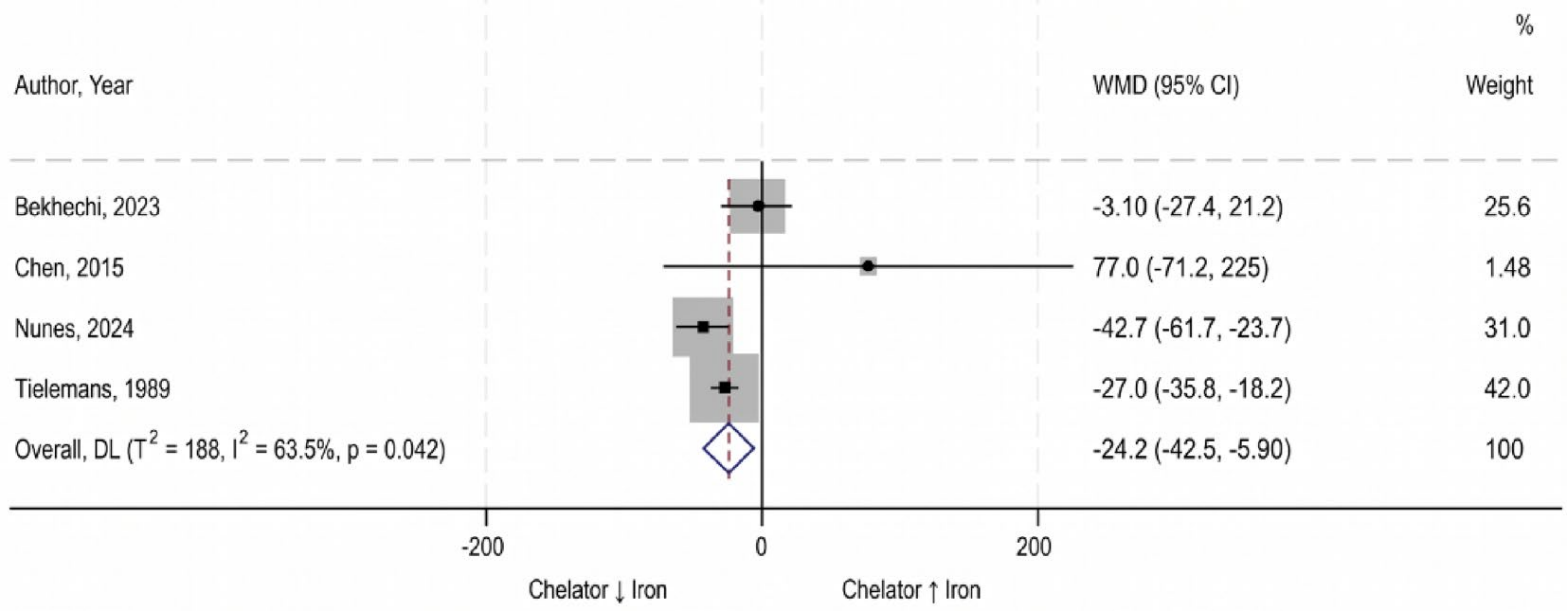
Forest plot of the effect of iron chelators on serum iron in CKD patients. Weights are from the random‐effects model.

### 3.4 Effect of Iron Chelators on Red Blood Cells Measures in CKD

#### 3.4.1 Effect of Iron Chelators on Hemoglobin in CKD

The meta‐analysis included five studies assessing the effect of iron chelators on hemoglobin levels in CKD patients [21, 22, 23, 28, 30] (Figure 5). The analysis compared the hemoglobin levels before and after the administration of iron chelators. The overall analysis demonstrated a positive effect of iron chelators on hemoglobin, with a significant WMD of 1.07 (95% CI: 0.20, 1.94). The overall analysis indicated high heterogeneity (*I* ^2^ = 74.6%), suggesting that there may be differences in the effect of iron chelators on hemoglobin between studies. The Doi plot revealed major asymmetry (LFK index = +3.68), suggesting potential risk of major publication bias (Figure S4).

**FIGURE 5:**
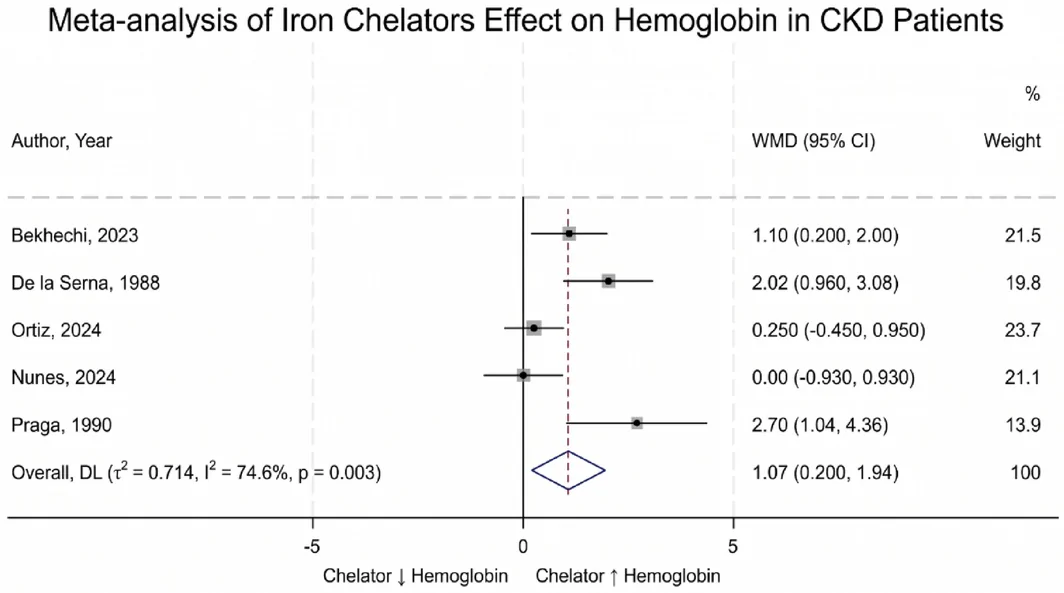
Forest plot of the effect of Iron chelators on hemoglobin in CKD patients. Weights are from the random‐effects model.

#### 3.4.2 Effect of Iron Chelators on Hematocrit in CKD

The meta‐analysis included four studies assessing the effect of iron chelators on hematocrit in CKD patients [21, 24, 26, 28] (Figure S5). The analysis compared the hematocrit percentages before and after the administration of iron chelators. The overall analysis demonstrated a positive effect of iron chelators on hematocrit, with a nonsignificant WMD of 1.98 (95% CI: −0.42, 4.37). The overall analysis indicated high heterogeneity (*I* ^2^ = 74.6%), suggesting that there may be differences in the effect of iron chelators on hematocrit between studies. The Doi plot revealed no asymmetry (LFK index = +0.27), suggesting no risk of publication bias (Figure S6).

### 3.5 Effect of Iron Chelators on Lipid Measures in CKD

#### 3.5.1 Effect of Iron Chelators on Cholesterol in CKD

Four studies reported the effect of iron chelators on cholesterol in patients with CKD [21, 23, 24, 25] (Figure S7). The analysis compared the serum cholesterol levels before and after the administration of iron chelators. The overall synthesis demonstrated no effect of iron chelators on serum cholesterol levels, with a WMD of −0.04 (95% CI: −4.80, 4.00). The overall analysis indicated no heterogeneity (*I* ^2^ = 0.0%). The Doi plot revealed no asymmetry (LFK index = +0.10), suggesting no risk of publication bias (Figure S8).

#### 3.5.2 Effect of Iron Chelators on Triglycerides in CKD

Four studies reported the effect of iron chelators on serum triglycerides in patients with CKD [21, 23, 24, 25] (Figure S9). The analysis compared the serum triglyceride levels before and after the administration of iron chelators. The overall synthesis demonstrated no effect of iron chelators on serum triglycerides' levels, with a WMD of −0.53 (95% CI: −8.42, 7.35). The overall analysis indicated no heterogeneity (*I* ^2^ = 0.0%). The Doi plot revealed major asymmetry (LFK index = −5.26), suggesting potential risk of major publication bias (Figure S10).

### 3.6 Effect of Iron Chelators on Glucose in CKD

Three studies reported the effect of iron chelators on serum glucose in patients with CKD [21, 24, 25] (Figure S11). The analysis compared the serum glucose levels before and after the administration of iron chelators. The overall synthesis demonstrated a reduction of serum glucose after administration of iron chelators, with a WMD of −5.79 (95% CI: −10.7, −0.87). The overall analysis indicated no heterogeneity (*I* ^2^ = 0.0%). The Doi plot revealed major asymmetry (LFK index = +4.15), suggesting potential risk of major publication bias (Figure S12).

### 3.7 Safety of Iron Chelators in CKD

Among the 11 included studies and the 206 studied patients, adverse effects were reported in varying numbers of patients from four studies [16, 23, 24, 30] (Table 2). A range of side effects was reported across the four included studies. The most common side effects were nausea (22 cases), abdominal pain (16 cases), and vomiting (nine cases), with higher frequencies observed in the study by Bekhechi et al. [23]. Other gastrointestinal issues, including diarrhea and constipation, were less frequent, reported in three and two cases, respectively. Less common side effects included skin rash (one case), chest pain (one case), and liver dysfunction (five cases). Notably, visual issues and acute pancreatitis were each reported in one case, highlighting the potential for more severe adverse events with iron chelator therapy. For a detailed breakdown of the side effects, refer to Table 2.

**TABLE 2:** Summary of adverse events in included studies.

Side effects	Bekhechi et al. [23]	Chen, Shu, and Yang [24]	de la Serna et al. [30]	Stivelman et al. [16]	Total
*N*	31	8	13	8	60
Nausea	19	3	—	—	22
Vomiting	6	3	—	—	9
Abdominal pain	12	4	—	—	16
Diarrhea	3	—	—	—	3
Constipation	—	2	—	—	2
Skin rash	—	1	—	—	1
Chest pain	—	1	—	—	1
Liver dysfunction	4	1	—	—	5
Visual issues	—	—	1	—	1
Acute pancreatitis	—	—	—	1	1

### 3.8 Quality and Risk of Bias Assessment

#### 3.8.1 Risk of Bias Assessment in Observational Studies

The methodological quality of the included observational studies was assessed using the NOS, with a visual summary of the risk of bias presented in Figure S13. In the “Selection” (D1) domain, Nunes et al. [22], Bekhechi et al. [23] and Tielemans et al. [29] were rated as low risk of bias, while the others had some concerns of bias in this domain due to potential limitations in participant selection or representativeness [16, 24, 25, 26, 28]. All studies were deemed low risk of bias in the “Comparability” (D2) domain, indicating appropriate control for confounding factors. Similarly, all studies were assessed as low risk in the “Outcome” (D3) domain, with clear outcome definitions and reliable measurement methods. Overall, all studies were categorized as having a low risk of bias.

#### 3.8.2 Risk of Bias Assessment in RCTs

In regard to the three RCTs, they were evaluated for risk of bias using the RoB 2.0 tool across five domains: bias arising from the randomization process (D1), deviations from intended interventions (D2), missing outcome data (D3), measurement of the outcome (D4), and selection of the reported result (D5). Overall, the studies showed mixed assessments, with some concerns regarding methodological rigor. Murillo Ortiz et al. [21] was judged to have a low risk of bias overall, with low risk in four domains and only some concerns in deviations from intended interventions. In contrast, De Marchi and Cecchin [27] and de la Serna et al. [30] were both rated as having some concerns overall. Concerns of bias in the randomization process, handling missing outcome data, and potential concerns about the selection of reported results were noted in de la Serna et al. [30]. De Marchi and Cecchin [27] lacked sufficient information on missing outcome data and exhibited concerns regarding selective reporting and deviation from the intended intervention. All three studies demonstrated low risk of bias in outcome measurement. While Murillo Ortiz et al. [21] provided strong methodological quality, the remaining studies highlight limitations that may affect confidence in their results. A detailed domain‐level assessment is presented in Figure S14.

## 4 Discussion

This systematic review and meta‐analysis represent the first comprehensive effort to evaluate the efficacy and safety of iron chelation therapy in patients with CKD. By synthesizing and analyzing data from three RCTs and eight observational studies involving 206 participants, we found consistent evidence that chelators, particularly deferasirox, reduce iron burden while improving selected hematological and metabolic parameters in CKD patients. Both deferasirox and deferoxamine significantly lowered serum transferrin and ferritin, with deferasirox producing a more homogeneous reduction in transferrin saturation (WMD −11.84 mg/dL, *I* ^2^ = 0%), whereas deferoxamine showed smaller and more variable effects (WMD −8.38 mg/dL, *I* ^2^ = 69.9%). Pooled analyses demonstrated a significant reduction in serum ferritin and serum iron. These findings establish iron chelators as effective agents for reducing systemic iron load in CKD, consistent with their established role in transfusion‐dependent anemias such as β‐thalassemia [31, 32].

Beyond iron indices, chelation therapy exerted beneficial effects on red cell parameters. Hemoglobin increased significantly across studies, while hematocrit showed a nonsignificant upward trend. This suggests that chelation not only reduces toxic iron deposition but may also restore erythropoietic function, potentially by lowering oxidative stress and preserving red cell survival. This aligns with Lee, Ho, and Tarng [33], who explained that chelation therapy's restorative effect on erythropoiesis is linked to inhibition of iron‐induced oxidative stress, reducing erythroid precursors' apoptosis, normalization of EPO‐regulating pathways, and mitigation of organ toxicity that otherwise suppresses red blood cell production and shortens red cell lifespan. Additionally, metabolic outcomes also demonstrated signal‐level effects. Interestingly, serum glucose declined modestly but consistently, whereas cholesterol and triglycerides remained unchanged. The glucose effect is particularly noteworthy given growing evidence that iron overload contributes to insulin resistance and metabolic dysfunction via oxidative stress pathways. This is consistent with Sobieska et al. [34], who illustrated that excess iron promotes the generation of reactive oxygen species (ROS) via the Fenton reaction, leading to oxidative stress that damages cellular components and disrupts insulin receptor phosphorylation. This oxidative environment impairs insulin signaling pathways by inhibiting key molecules such as IRS‐1 and AKT, reducing glucose uptake in muscle and liver cells, while simultaneously activating stress kinases like JNK, thereby further aggravating insulin resistance. These findings raise the possibility that iron chelation may offer additional metabolic benefits in CKD beyond iron removal.

Adverse effects were mostly gastrointestinal, including nausea, abdominal pain, and vomiting, with higher frequencies in recent deferasirox studies. Rare but clinically important complications such as liver dysfunction, visual disturbances, and acute pancreatitis were also reported. This profile mirrors experience in non‐CKD populations, though nephrotoxicity remains a theoretical concern in CKD, given pre‐existing renal impairment [35]. Consistent with broader clinical use, iron chelators have been associated with agent‐specific toxicities, including ocular and auditory disturbances with deferoxamine, gastrointestinal and renal events with deferasirox, and agranulocytosis with deferiprone [36]. This emphasizes the importance of careful agent selection and monitoring.

A recent review by Nashwan and Yassin [17] highlighted both the promise and challenges of deferasirox use in patients with CKD on dialysis, noting its efficacy in reducing iron overload and the advantage of oral administration, but also emphasizing limited CKD‐specific data, potential renal and metabolic adverse effects, drug interactions, and cost concerns. Our meta‐analysis adds quantitative evidence to this largely narrative field, helping to clarify the efficacy and safety profile of chelation in this unique patient group.

Our results in CKD concur with findings from meta‐analyses in thalassemia populations, such as Dou et al. [37], which showed that deferasirox and deferoxamine achieve broadly comparable efficacy when dosed appropriately, though deferasirox offers greater convenience at the expense of more gastrointestinal and renal adverse events. However, CKD introduces confounding variables like chronic inflammation, impaired erythropoiesis, and frequent parenteral iron administration, none of which have been synthesized before. By extending this evidence base into CKD, our study highlights that similar therapeutic balances between efficacy and safety may apply in patients with renal impairment, elucidating the importance of careful agent selection and monitoring in this vulnerable group.

Our work also complements earlier meta‐analyses of metal chelation in CKD, such as the study by Yang et al. [38], which demonstrated that calcium disodium EDTA slowed CKD progression in patients with elevated body lead burdens by improving eGFR and creatinine clearance. Collectively, these findings suggest that targeted chelation, whether for heavy metals like lead or for iron overload, may play a broader reno‐protective role in CKD, although the specific agents, mechanisms, and patient populations differ.

The heterogeneity observed across outcomes likely reflects both clinical and methodological variability. Key clinical sources include dialysis modality, baseline iron burden, ESA exposure, and adherence to chelation regimens. Methodological and population characteristics contributors include small sample sizes, variable treatment durations (6–84 months), and heterogeneity in drug type and route of administration (oral vs. intravenous/intradialytic). This reinforces that the observed variability is driven more by outcome‐ and setting‐specific factors than by the chelator class itself. Notably, Chen, Shu, and Yang [24] reported paradoxical increases in serum iron, which contributed to between‐study inconsistency. Despite these limitations, the overall direction of effect remained robust, strengthening confidence in the therapeutic potential of chelation in CKD.

This study's findings are biologically plausible and align with mechanistic evidence. By binding and mobilizing catalytic iron, chelators minimize the catalytic iron pool responsible for ROS generation, mitigating oxidative damage to tissues and red cells. This reduction in oxidative stress likely underpins improvements in hemoglobin and the modest glucose‐lowering effects observed [39, 40]. Experimental data further support a link between iron overload and metabolic dysfunction, suggesting a broader role for chelation in improving outcomes beyond anemia correction [41].

The strengths of this meta‐analysis lie in its novelty, clinical relevance, and comprehensive scope. It is the first evidence‐based quantitative synthesis of iron chelation therapy in CKD, incorporating data from 11 studies across multiple countries, dialysis modalities, and chelator types to comprehensively assess both efficacy and safety outcomes. The consistent overall direction of effect across outcomes strengthens confidence in the therapeutic potential of chelation, while the analysis addresses a critical knowledge gap by focusing on a population with unique risk factors for iron overload.

Nonetheless, several limitations warrant consideration. Our analysis includes only 11 studies (206 patients), mostly observational with three randomized trials, limiting statistical power and generalizability. Studies varied in design, duration, and population across multiple countries, contributing to significant heterogeneity in ferritin, hemoglobin, hematocrit, and serum iron outcomes—particularly for deferoxamine. Despite these constraints, the consistent direction of effect across outcomes strengthens confidence that observed benefits are not due to chance. This synthesis is novel in focusing specifically on CKD, distinct from the hematology literature on thalassemia where most chelation evidence originates. Accordingly, pooled estimates should be interpreted as hypothesis‐generating rather than definitive. Additionally, incomplete adverse event reporting and absence of long‐term clinical outcomes preclude robust safety and broader impact assessment.

Despite these limitations, the findings have clear implications for clinical practice. For CKD patients with iron overload, particularly those unable to undergo phlebotomy due to anemia, chelation, especially with deferasirox, offers a viable option. It helps mitigate iron toxicity, improve hematologic outcomes, and potentially confer metabolic benefits. However, treatment should be individualized, with careful monitoring for gastrointestinal, hepatic, and renal adverse effects. Future research should prioritize adequately powered, pragmatic randomized trials to establish definitive efficacy and safety, define optimal dosing strategies, and evaluate long‐term outcomes. Mechanistic studies in humans linking iron overload to oxidative stress, inflammation, and metabolic dysfunction in CKD will further clarify the broader potential of chelation therapy in this population.

## 5 Conclusion

In conclusion, iron chelation therapy effectively reduces iron overload and improves hemoglobin in CKD patients, with deferasirox showing consistent transferrin reduction and deferoxamine demonstrating greater ferritin decline, albeit with higher variability. A modest but significant drop in serum glucose suggests potential metabolic benefits, likely through reduced oxidative stress, while lipids remained unaffected. Gastrointestinal side effects were common; rare but serious toxicities warrant vigilant monitoring. Despite limited high‐quality evidence and substantial heterogeneity, the consistency of effects across diverse populations supports chelation as a viable option for iron‐overloaded CKD patients, particularly where phlebotomy is not feasible. Larger, long‐term randomized trials are urgently needed to confirm clinical benefits, optimize dosing, and evaluate hard outcomes like mortality and cardiovascular risk.

## Funding

The authors have nothing to report.

## Consent

The authors have nothing to report.

## Conflicts of Interest

The authors declare no conflicts of interest.

## Supporting information


**Table S1:** Detailed search strategy for each database.
**Figure S1:** Doi plot assessing publication bias in the meta‐analysis of transferrin.
**Figure S2:** Doi plot assessing publication bias in the meta‐analysis of ferritin.
**Figure S3:** Doi plot assessing publication bias in the meta‐analysis of serum iron.
**Figure S4:** Doi plot assessing publication bias in the meta‐analysis of hemoglobin.
**Figure S5:** Forest plot showing a comparison of mean hematocrit levels before and after iron chelation.
**Figure S6:** Doi plot assessing publication bias in the meta‐analysis of hematocrit.
**Figure S7:** Forest plot showing a comparison of mean cholesterol levels before and after iron chelation.
**Figure S8:** Doi plot assessing publication bias in the meta‐analysis of cholesterol.
**Figure S9:** Forest plot showing a comparison of mean triglyceride levels before and after iron chelation.
**Figure S10:** Doi plot assessing publication bias in the meta‐analysis of triglycerides.
**Figure S11:** Forest plot showing a comparison of mean glucose levels before and after iron chelation.
**Figure S12:** Doi plot assessing publication bias in the meta‐analysis of glucose.
**Figure S13:** Newcastle–Ottawa Scale (NOS) quality assessment for included observational studies.
**Figure S14:** Risk of Bias 2.0 (RoB 2.0) assessment for included randomized controlled trials.

## Data Availability

The data that support the findings of this study are available from the corresponding author upon reasonable request.
