# Supplementary material for: Safety and Efficacy of Iron Chelators in Patients With Chronic Kidney Disease: A Systematic Review and Meta‐Analysis
Source: Hemodial Int. 2026 Jun 18;30(4):745–56. doi: 10.1111/hdi.70103 (PMC13638482; doi:10.1111/hdi.70103)
Supplement: Supplementary file 1 [file HDI-30-745-s001.docx]

**Supplementary Material**

**Supplementary Table S1. Detailed Search Strategy**

| **Database** | **Iron Chelating Agents Search Strategy** | **Chronic Kidney Disease / Dialysis Search Strategy** | **Results (23/06/2025)** |
| --- | --- | --- | --- |
| **PubMed** | ((Iron Chelating Agents[MeSH Terms]) OR (deferoxamine[Title/Abstract]) OR (deferiprone[Title/Abstract]) OR (deferasirox[Title/Abstract])) | ((Kidney Failure, Chronic[MeSH Terms]) OR (chronic kidney disease[Title/Abstract]) OR (chronic renal disease[Title/Abstract]) OR (chronic kidney failure[Title/Abstract]) OR (chronic renal failure[Title/Abstract]) OR (end stage renal disease[Title/Abstract]) OR (end-stage renal disease[Title/Abstract]) OR (end stage renal failure[Title/Abstract]) OR (end-stage renal failure[Title/Abstract]) OR (end stage kidney failure[Title/Abstract]) OR (end-stage kidney failure[Title/Abstract]) OR (CKD[Title/Abstract]) OR (CKF[Title/Abstract]) OR (ESRD[Title/Abstract]) OR (ESKD[Title/Abstract]) OR (Renal Dialysis[MeSH Terms]) OR (Renal Replacement Therapy[MeSH Terms])) | 251 |
| **Cochrane CENTRAL** | MeSH descriptor: [Iron Chelating Agents] explode all trees OR ((deferoxamine) OR (deferiprone) OR (deferasirox)):ti,ab,kw | MeSH: [Kidney Failure, Chronic] OR [Renal Dialysis] OR [Renal Replacement Therapy] OR text terms (CKD, ESRD, etc.) in ti,ab,kw | 44 |
| **CINAHL** | XB (MH "Deferoxamine" OR (deferoxamine OR deferiprone OR deferasirox)) | XB ((MM "Kidney Failure, Chronic+") OR (MM "Dialysis+") OR (“chronic kidney disease” OR “chronic renal disease” OR “chronic kidney failure” OR “chronic renal failure” OR “end stage renal disease” OR “end-stage renal disease” OR “end stage renal failure” OR “end-stage renal failure” OR “end stage kidney failure” OR “end-stage kidney failure” OR CKD OR CKF OR ESRD OR ESKD)) | 21 |
| **Scopus** | TITLE-ABS("iron chelator" OR "iron chelating agent" OR "deferoxamine" OR "deferiprone" OR "deferasirox") | TITLE-ABS("chronic kidney disease" OR "chronic renal disease" OR "chronic kidney failure" OR "chronic renal failure" OR "end stage renal disease" OR "end-stage renal disease" OR "end stage renal failure" OR "end-stage renal failure" OR "end stage kidney failure" OR "end-stage kidney failure" OR CKD OR CKF OR ESRD OR ESKD OR dialysis OR hemodialysis OR "peritoneal dialysis" OR "renal replacement therapy") | 266 |
| **EMBASE** | 'iron chelating agent'/exp OR ((iron AND chelator) OR (iron AND chelating AND agent) OR deferoxamine OR deferiprone OR deferasirox):ti,ab | ('chronic kidney failure'/exp OR 'hemodialysis'/exp OR (((chronic AND kidney AND disease) OR (chronic AND renal AND disease) OR (chronic AND kidney AND failure) OR (chronic AND renal AND failure) OR (end AND stage AND renal AND disease) OR ('end stage' AND renal AND disease) OR (end AND stage AND renal AND failure) OR ('end stage' AND renal AND failure) OR (end AND stage AND kidney AND failure) OR ('end stage' AND kidney AND failure) OR ckd OR ckf OR esrd OR eskd):ti,ab) | 452 |
| **Web of Science** | TI=((Iron Chelating Agents) OR (Iron Chelator) OR (deferoxamine) OR (deferiprone) OR (deferasirox)) OR AB=((Iron Chelating Agents) OR (Iron Chelator) OR (deferoxamine) OR (deferiprone) OR (deferasirox)) | TI=((chronic kidney disease) OR (chronic renal disease) OR (chronic kidney failure) OR (chronic renal failure) OR (end stage renal disease) OR (end-stage renal disease) OR (end stage renal failure) OR (end-stage renal failure) OR (end stage kidney failure) OR (end-stage kidney failure) OR (CKD) OR (CKF) OR (ESRD) OR (ESKD) OR (Renal Dialysis) OR (Renal Replacement Therapy)) OR AB=((chronic kidney disease) OR (chronic renal disease) OR (chronic kidney failure) OR (chronic renal failure) OR (end stage renal disease) OR (end-stage renal disease) OR (end stage renal failure) OR (end-stage renal failure) OR (end stage kidney failure) OR (end-stage kidney failure) OR (CKD) OR (CKF) OR (ESRD) OR (ESKD) OR (Renal Dialysis) OR (Renal Replacement Therapy)) | 143 |

**Supplementary Figure S1. Forest plot of the effect of Iron chelators on transferrin in CKD patients**

**Supplementary Figure S2. Doi plot of the effect of Iron chelators on ferritin in CKD patients**

**Supplementary Figure S3. Doi plot of the effect of Iron chelators on serum iron in CKD patients**

**Supplementary Figure S4. Doi plot of the effect of Iron chelators on hemoglobin in CKD patients**

**Supplementary Figure S5:** **Forest plot of the effect of Iron chelators on hematocrit in CKD patients**

**Supplementary Figure S6. Doi plot of the effect of Iron chelators on hematocrit in CKD patients**

**Supplementary Figure S7:** **Forest plot of the effect of Iron chelators on cholesterol in CKD patients**

**Supplementary Figure S8. Doi plot of the effect of Iron chelators on cholesterol in CKD patients**

**Supplementary Figure S9:** **Forest plot of the effect of Iron chelators on triglycerides in CKD patients.**

**Supplementary Figure S10. Doi plot of the effect of Iron chelators on triglycerides in CKD patients.**

**Supplementary Figure S11:** **Forest plot of the effect of Iron chelators on glucose in CKD patients.**

**Supplementary Figure S12. Doi plot of the effect of Iron chelators on glucose in CKD patients.**

**Supplementary Figure S13. New Castle Ottawa Quality Assessment of Observational Studies**

**Supplementary Figure S14. New Castle Ottawa Quality Assessment of Observational Studies**
